# Pathogenesis to management of hepatocellular carcinoma

**DOI:** 10.18632/genesandcancer.226

**Published:** 2022-12-13

**Authors:** Ben L. Da, Kelly I. Suchman, Lawrence Lau, Atoosa Rabiee, Aiwu Ruth He, Kirti Shetty, Herbert Yu, Linda L. Wong, Richard L. Amdur, James M. Crawford, Sharon S. Fox, Gregory M. Grimaldi, Priya K. Shah, Jonathan Weinstein, David Bernstein, Sanjaya K. Satapathy, Nyasha Chambwe, Xiyan Xiang, Lopa Mishra

**Affiliations:** ^1^Department of Internal Medicine, Division of Hepatology, Sandra Atlas Bass Center for Liver Diseases and Transplantation, Donald and Barbara Zucker School of Medicine at Hofstra/Northwell Health, Manhasset, NY 11030, USA; ^2^Department of Internal Medicine, Donald and Barbara Zucker School of Medicine at Hofstra/Northwell Health, Manhasset, NY 11030, USA; ^3^Department of Surgery, North Shore University Hospital, Northwell Health, Manhasset, NY 11030, USA; ^4^Department of Gastroenterology and Hepatology, VA Medical Center, Washington, DC 20422, USA; ^5^Lombardi Comprehensive Cancer Center, Georgetown University Medical Center, Washington, DC 20007, USA; ^6^Division of Gastroenterology and Hepatology, University of Maryland, Baltimore, MD 21201, USA; ^7^Department of Epidemiology, University of Hawaii Cancer Center, Honolulu, HI 96813-5516, USA; ^8^Department of Surgery, University of Hawaii, Honolulu, HI 96813-5516, USA; ^9^Quantitative Intelligence, The Institutes for Health Systems Science and Bioelectronic Medicine, The Feinstein Institutes for Medical Research, Northwell Health, Manhasset, NY 10022, USA; ^10^Department of Pathology and Laboratory Medicine, Donald and Barbara Zucker School of Medicine at Hofstra/Northwell, Hempstead, NY 11549, USA; ^11^Department of Radiology, Northwell Health, Donald and Barbara Zucker School of Medicine at Hofstra/Northwell, Manhasset, NY 11030, USA; ^12^Division of Vascular and Interventional Radiology, Department of Radiology, Northwell Health, Donald and Barbara Zucker School of Medicine at Hofstra/Northwell, Manhasset, NY 11030, USA; ^13^The Institute of Molecular Medicine, The Feinstein Institutes for Medical Research, Northwell Health, Manhasset, NY 11030, USA; ^14^The Institute for Bioelectronic Medicine, The Feinstein Institutes for Medical Research and Cold Spring Harbor Laboratory, Department of Medicine, Division of Gastroenterology and Hepatology, Northwell Health, Manhasset, NY 11030, USA

**Keywords:** pathogenesis, HCC management, genomic heterogeneity, targeted therapy

## Abstract

Hepatocellular carcinoma (HCC) is the most common primary liver cancer whose incidence continues to rise in many parts of the world due to a concomitant rise in many associated risk factors, such as alcohol use and obesity. Although early-stage HCC can be potentially curable through liver resection, liver-directed therapies, or transplantation, patients usually present with intermediate to advanced disease, which continues to be associated with a poor prognosis. This is because HCC is a cancer with significant complexities, including substantial clinical, histopathologic, and genomic heterogeneity. However, the scientific community has made a major effort to better characterize HCC in those aspects via utilizing tissue sampling and histological classification, whole genome sequencing, and developing viable animal models. These efforts ultimately aim to develop clinically relevant biomarkers and discover molecular targets for new therapies. For example, until recently, there was only one approved systemic therapy for advanced or metastatic HCC in the form of sorafenib. Through these efforts, several additional targeted therapies have gained approval in the United States, although much progress remains to be desired. This review will focus on the link between characterizing the pathogenesis of HCC with current and future HCC management.

## INTRODUCTION

**Figure 1:**
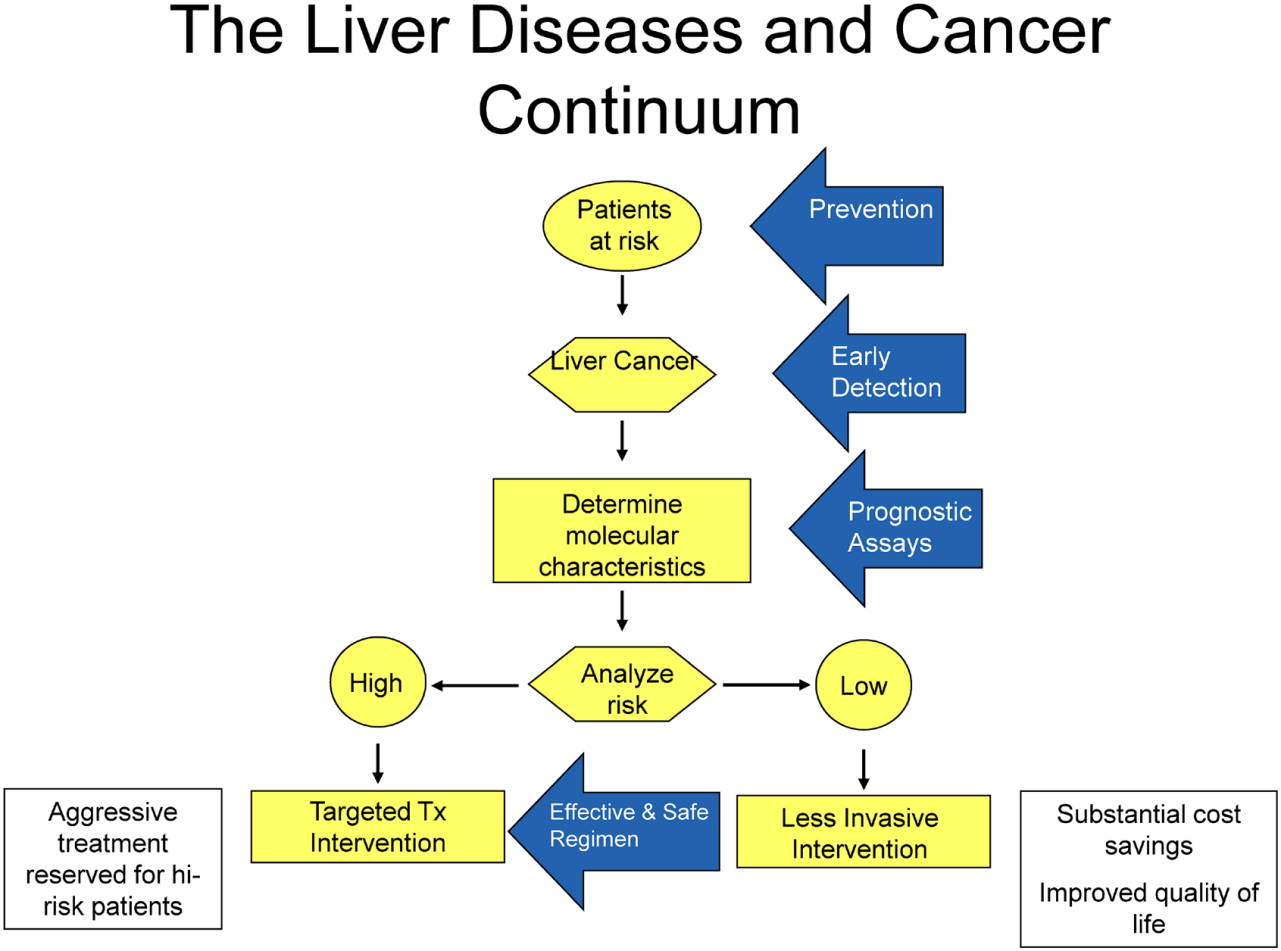
The liver diseases and cancer continuum. Advances in the prevention, early detection, and prognostication of HCC through epidemiological studies, histological classification, genomics, biomarkers, and animal models will enable risk stratification and improved patient management.

**Figure 2:**
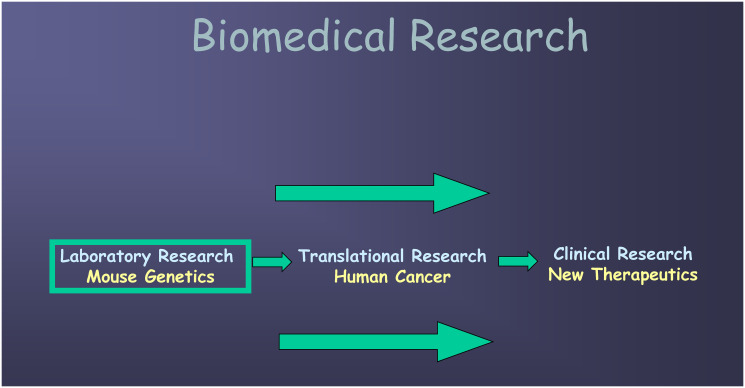
Laboratory research to clinical research pathway. The goal is to start with laboratory research in mouse models, translate relevant findings to human hepatocellular carcinoma, and finally arrive at testable new clinical therapeutics that will impact future cancer management.

Hepatocellular carcinoma (HCC) is the sixth most common cancer worldwide and the third most common cause of cancer death [1]. The rate of HCC continues to rise, especially in the United States and Central America [2]. Worldwide, the number of new primary liver cancer cases among men and women in 2020 was 577,522 and 252,658, respectively [1]. Most cases are diagnosed in the advanced stages. The presumed reason for the increasing rate of HCC is the rising incidence of liver disease related to alcohol overuse and metabolic syndrome, associated with a dramatic rise in obesity and diabetes mellitus [3–6]. New approaches harnessing large-scale epidemiological studies, histological classification, genomics, biomarkers, and animal models have provided new insights into the development of HCC. These advances are expected to alter the future of HCC management (Figures 1 and 2). This review will focus on the link between the pathogenesis and management of HCC.

### Tissue is the issue

The development of HCC typically follows a path from liver inflammation and fibrosis toward a disordered nodular liver architecture (cirrhosis), with the potential progression of nodules through dysplasia to cancer [7]. Dysplastic nodules are further classified into low and high-grade, with high-grade nodules having a much higher risk of progressing to HCC. Once malignant, HCC becomes capable of invasion into adjacent areas of the liver and, more ominously, invasion into the vasculature with the potential for widespread dissemination [8]. There are multiple morphological subtypes of HCC, including biphenotypic (combined HCC and CCA), cirrhotomimetic (cancer cells infiltrating many parts of a lobe or the entire liver), and more. These are depicted in Table 1 [9]. HCC is the major type of primary liver cancer. In contrast, another type, cholangiocarcinoma (CCA), develops from the bile ducts. When present in the liver, CCA is referred to as intrahepatic CCA and represents 5–10% of all liver cancer cases. Distinguishing between CCA and HCC is critically important as the difference determines the course of treatment [10].

**Table 1:** Hepatocellular carcinoma subtypes and prognosis

Subtype	Frequency in Surgical Pathology Specimens (%)	Prognosis (compared with conventional HCC)
Steathohepatic	20	Similar
Clear Cell	7	Better
Scirrhous	4	Similar to better
Cirrhotomimetic	1	Worse
Combined hepatocellular cholangiocarcinoma	1	Worse
Granulocyte colony-stimulating factor producing	1	Similar to better
Sarcomatoid	<1	Worse
Carcinosarcoma	<1	Worse
Carcinoma with osteoclast-like giant cells	<1	Worse
Lymphocyte rich	<1	Better
Provisional subtypes		
Chromophobe	1–2	Unclear
Combined hepatocellular cholangiocarcinoma with stem cell features	<1	Unclear
Lipid rich	<1	Unclear
Myxoid	<1	Unclear
Syncytial giant cell	<1	Unclear
Transitional cell	<1	Unclear

Traditionally, the diagnosis of HCC was primarily made with characteristic findings on contrast-enhanced imaging, as tumor biopsies were done infrequently due to a risk of bleeding and needle-track seeding of the tumor [11]. However, tissue sampling is again indicated owing to the need for histological classification and genomic testing to select targeted therapy, guide management, and provide an informed prognosis. The development of coaxial needle techniques has resulted in negligible risks of tumor dissemination [12, 13], leading to greater acceptance of tumor biopsies. Recently, there has been increasing interest in the molecular analysis of tumor-derived circulating tumor cells (CTCs), extracellular vesicles (EVs), and cell-free circulating nucleic acids (cfDNA or cfRNA), termed “liquid biopsy” [14]. Although these approaches might be complementary, particularly when tissue samples are insufficient or unsuitable for biomarker testing [15], tissue-based analysis remains the preferred method for molecular testing when tissue is available [16].

### Genetic alterations in HCC

Human HCC has significant clinical, histopathologic, and genomic heterogeneity. Cancer cells can vary from well to poorly differentiated and may arise from multiple cell lines [17]. For example, cancer stem cells (that self-renew and can also generate various progeny) are believed to give rise to nearly 40% of HCCs. They may be one of the factors responsible for tumor heterogeneity and resistance to therapy [18, 19]. In addition, multiple genetic events have been associated with the development and pathogenesis of HCC, including mutations, amplifications, deletions, chromosomal rearrangements, and aberrant methylation [20]. Advancements in our understanding of these events guide the development of therapies based on the presence of individual or groups of genetic alterations [21]. We will now review some of the genetic alterations associated with the pathogenesis of HCC.

The most common mutations in HCC are somatic mutations in the promoter of the gene encoding telomerase reverse transcriptase (*TERT*), which are present in 54–60% of all HCC cases [22, 23]. Telomeres protect chromosome ends and become shorter with repeated cell division, while telomerase is the enzyme responsible for maintaining the length of the telomere. Telomere shortening is accentuated in chronic liver injury, resulting in apoptosis and the inability of the liver to regenerate fully, and is likely linked to the pathogenesis of HCC [11].

HCC is also associated with a high frequency of mutations in several other genes. A standard mutational profile, present in 31% of HCCs, is inactivating mutations of *TP53*, encoding the tumor-suppressor p53. Four other common mutations in HCC activate WNT pathway genes (*CTNNB1*, *AXIN 1/2*, *APC*; 20% of cases) [24, 25]. Other mutations alter a chromatin remodeling pathway (*ARID1A* and *ARID2*; 7% and 5% of cases, respectively), while others affect a gene encoding a deubiquitinase (*BAP1*; 5%) [22, 24, 25].

Calderaro et al. analyzed mutations associated with HCC and correlated the genetic mutations present with their clinical presentation [24]. Tumors with *CTNNB1* mutations were large, well-differentiated, cholestatic, and lacked markers of inflammation. Meanwhile, tumors with *TP53* mutations were poorly differentiated, multinucleated, and exhibited frequent vascular invasion. Patients with *TERT* promoter mutations tend to be older, predominantly male, and more likely to be HCV positive. In addition, mutations in the TGF-β pathway occur in up to 40% of HCCs. Alterations in the TGF-β pathway can be characterized by elevated *HMGA2* and *TERT* levels, highlighting the concept of how many pathways can interact to drive HCC [23].

Gene amplification and deletions, as well as chromosomal rearrangements, are structural alterations that play an important role in the carcinogenesis of HCC. Amplification of the genes *CCND1* (encoding cyclin D1) was present in 15% of the HCCs studied in one report [25]. Common deletions found in HCC include those found in *CDKN2A* (encoding both the proteins p16 and p14, tumor suppressors) or *RB1* (encoding the tumor suppressor retinoblastoma transcriptional corepressor 1) [19]. Furthermore, a chromosomal rearrangement involving chromosome 19 resulting in the formation of chimeric RNAs is associated with fibrolamellar HCC [26].

Epigenetic alterations include changes in methylation, chromatin remodeling, and micro-RNAs. Aberrant methylation of multiple promoters is associated with advanced HCC, chronic viral hepatitis B or C, and liver cirrhosis [20, 27]. Recent reports indicate that changes in 19 miRNAs correlated with disease outcomes, suggesting that this miRNA signature may be used as a disease prognosticator [28].

### The potential of animal models

Animal models allow for the integration of the genomic and molecular characterization of human HCC samples in a way that permits for the discovery and testing of biomarkers and novel HCC therapies. Examples of animal models that have been developed which resemble liver cancer in humans include the *C-Myc/p53*, *Smad4/PTEN* (CCA), *PDGFR*, *TAK1*, compromised TGF-β signaling, and *MUP-uPA* animal models [29–37]. Other animal models that have been developed include a mouse model of NASH and a human stem cell disease that exhibits spontaneous liver cancer and alcohol-induced enhancement for the development of liver cancer [29].

The NASH-HCC model is MUP-uPA mice fed a high-fat diet (HFD), resulting in steatohepatitis resembling human NASH. Its progression to HCC represents a reliable model of NASH-driven HCC that has been used to evaluate HCC-targeting immunotherapies. Cancer stem cell and alcohol-sensitive models are found in mice with compromised TGF-β signaling (*Smad2*^+/−^/*Smad3*^+/−^ mice) [30, 38]. In both cases, these are immune-competent models that can provide opportunities to investigate the crosstalk between cancer cells, their microenvironment, and the immune system, as well as explore potential contributions of the microbiome. Such information is critical for helping us understand the molecular causes responsible for the progression from liver injury to HCC, identify new therapeutic targets, design effective combination strategies and treatment regimens, and determine prevention strategies.

### Surveillance

Surveillance using abdominal ultrasound with or without serum alpha-fetoprotein (AFP) levels every six months is recommended by current guidelines for patients with cirrhosis and has been associated with improved survival [39, 40]. Certain populations without cirrhosis, such as those with hepatitis B, are also recommended to undergo HCC screening (depending on age and race) due to an elevated risk of HCC [41].

However, the effectiveness of HCC surveillance in clinical practice is severely limited by a lack of sensitivity and specificity of both ultrasound (particularly lesions under 1 cm) and AFP tests [42]. Ultrasound is also operator-dependent, and sensitivity can depend on the patient’s body habitus. These screening methods are underutilized as only 16.9% of primary care physicians and 51.7% of subspecialty physicians screen correctly per current liver guideline recommendations [43–47]. Finally, approximately 20% of HCCs arise in those without established cirrhosis creating further screening difficulties [48].

Contrast-enhanced computerized tomography (CT) and magnetic resonance imaging (MRI) scans offer better HCC detection and have traditionally allowed for the ability to make a diagnosis. However, the use of these imaging studies is limited by cost, insurance coverage, use of intravenous contrast, exposure to radiation, and claustrophobia [44, 46]. MRI is superior to CT in detecting HCC (particularly for small tumors) in this setting [49].

Due to the limitations of AFP as an HCC screening biomarker, novel biomarkers such as AFP-L3%, des-gamma-carboxy prothrombin (DCP), osteopontin, glycosylated proteins, and circulating tumor cells have been discovered and studied as individual and combination testing [50]. However, all these tests have significant flaws, such as AFP-L3%, which is hindered by low sensitivity in early disease [51]. Attempts have been made to combine biomarkers with clinical factors, such as the GALAD score incorporating age, gender, AFP, AFP-L3%, and DCP [52]. While this score improved the detection abilities of each biomarker alone, it is prone to false positives [52]. Recently, a promising blood-based panel of methylated DNA and protein markers has demonstrated improved early HCC detection capabilities (71% sensitivity and 90% specificity) compared to the GALAD score or AFP alone [53].

Future research should focus on precision screening for HCC considering patient characteristics and the risk of HCC development. Similarly, preventative approaches that eliminate HCC risk factors such as liver disease drivers (e.g., alcohol, HBV, metabolic syndrome, etc.) can substantially reduce rates of HCC [54]. Dietary and pharmacologic approaches to reducing the risk for HCC are also under consideration. Still, their role in clinical management needs to be further validated and defined [55–59].

### Treatment of early and intermediate stage HCC

**Figure 3:**
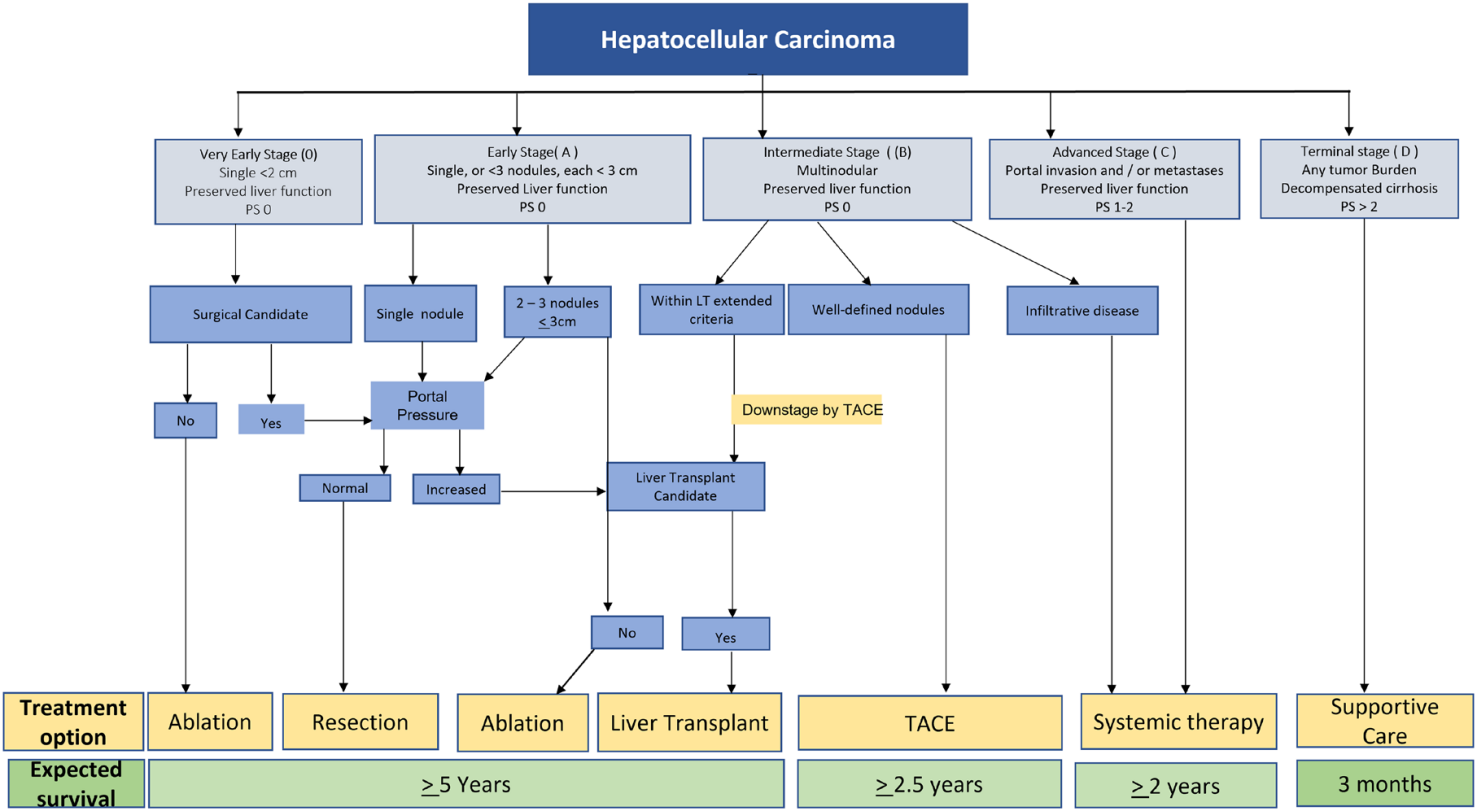
Barcelona clinic liver cancer (BCLC): update on staging, prognostication and treatment. Adapted from: Reig M, Forner A, Rimola J, Ferrer-Fàbrega J, Burrel M, Garcia-Criado A,Kelley RK et al. BCLC strategy for prognosis prediction and treatment recommendation: The 2022 update. J of Hepatology 2022; 76:681–93. Abbreviations: LT: liver transplant; PS: Eastern Cooperative Oncology Group performance status; TACE: transarterial chemoembolization.

Once diagnosed, treatment of HCC remains primarily based on the Barcelona Clinic Liver Cancer (BCLC) staging (Figure 3) [60, 61]. Individual academic centers often have multidisciplinary tumor boards where care can be discussed and treatment plans formulated. Curative therapy can be achieved for early (usually less than 3 cm) HCC via ablation or surgical resection for eligible patients. When possible, surgery offers the best overall and disease-free survival in the treatment of HCC. Surgical resection is generally limited to patients with a solitary tumor confined to the liver in the setting of well-compensated Child-Pugh Class A cirrhosis without significant portal hypertension and sufficient liver remnant volume after resection. In these selected patients, surgical resection has comparable survival to liver transplantation without the disadvantages of waiting for a suitable donor liver and the need for post-transplant immunosuppression [62, 63].

The key considerations in deciding between surgical resection and liver transplantation include an assessment of tumor stage, location, the severity of liver disease, and availability of donor liver grafts. Liver transplantation completely removes both the tumor and the diseased, tumorigenic liver. Due to the limited supply of donor livers and the need to share donor livers with non-HCC patients, the current Organ Procurement and Transplantation Network (OPTN) policy limits the eligibility of transplant candidates to those with T2 HCC lesions (a solitary lesion measuring 2 to 5 cm, or up to three lesions measuring 1 to 3 cm). This was based on excellent actuarial overall (75%) and recurrence-free survival (83%) observed in patients transplanted with HCC within “Milan Criteria” [64].

The allocation of donor livers is based on the Model for End-stage Liver Disease-sodium (MELD- Na) score. Because HCC patients often have relatively low MELD-Na scores compared to those with end-stage cirrhosis, these patients can be granted “exception points” depending on tumor size/number and after six months of waiting on the list (Table 2) [64]. The criteria for liver transplantation continue to evolve to balance the risk of death in end-stage cirrhosis patients with the risk of tumor progression in HCC patients. Liver-directed therapies (LDT) can be utilized to bridge patients to transplant or to “down-stage” HCC that is outside of Milan criteria but within “UCSF criteria” for liver transplantation (Table 2) [64, 65]. Recently, the important role of biomarkers in liver transplantation has been recognized with high AFP (>1000 ng/mL) used as a surrogate for aggressive tumor biology and disqualifying patients for MELD exception points. As the transplant criteria for HCC evolve, the future likely lies in utilizing a combination of disease prognosticating biomarkers, tumor size and number, and response to LDT [66].

**Table 2:** Liver transplant criteria

Milan Criteria	UCSF^*^ Criteria
· Single tumor ≤ 5 cm, or	· Single tumor ≤ 5 cm, or
· 2–3 tumors, none exceeding 3 cm	· 2–3 lesions, none exceeding 4.5 cm, with total tumor diameter ≤ 8 cm
· No vascular invasion and/or extrahepatic spread	· No vascular invasion and/or extrahepatic spread

^*^University of California, San Francisco.

Liver-directed therapies (LDT) can be used for curative intent, bridge to transplant, downstage to transplant, and palliative purposes. Ablation, bland embolization, transarterial chemoembolization (TACE), drug-eluting bead transarterial chemoembolization (DEB-TACE), and Yttrium90 transarterial radioembolization (TARE) are therapies to treat HCC. Ablation is considered the primary treatment for very early-stage HCC (BCLC 0) not amenable for resection or transplant. If ablation is not feasible, transarterial treatments such as TACE, DEB-TACE, and TARE are performed with notably high efficacy at high radiation doses administered during TARE [67]. This treatment approach applies to early-stage HCC (BCLC Stage A) patients and patients who are not surgical candidates. Ablation is less effective for larger masses, and transarterial treatments are usually the preferred treatment for those patients.

TACE has been the standard of care in intermediate-stage HCC (BCLC stage B) based on improvements in overall survival (OS) compared to supportive care alone in two randomized studies conducted before the approval of sorafenib. No high-level evidence shows improved survival of TACE compared to DEB-TACE. However, a recent phase 2 randomized controlled study (TRACE) demonstrated improved median overall survival and superior tumor control with TARE compared to DEB-TACE in BCLC Stage A and B patients who were not surgical or ablation candidates [68].

### Predicting surgical outcomes based on molecular alterations

Several studies have examined the correlation between genetic changes within tumor and non-tumor tissue and outcomes following hepatic resection and orthotopic liver transplantation (OLT). Mutations in *RB1* and *TP53* have been associated with an increased risk of tumor recurrence and decreased survival. Transcriptome signatures from tumor tissue correlate with tumor aggressiveness and early recurrence. Expression levels of 5 specific genes (5-gene score) in tumor tissue have been shown to correlate with early tumor recurrence and overall survival in patients undergoing hepatic resection [69]. An HCC subtype characterized by *TP53* mutation, high fractional allelic loss, significant global hypomethylation, and absence of *CTNNB1* mutation were noted to predict shorter recurrence-free survival in patients who underwent liver transplantation [70]. Whole transcriptomic profiling of gene expression signatures has identified progenitor cell markers as predictors of recurrence after OLT [71]. However, these studies are based on pathological analysis of the explanted liver and, therefore, cannot be utilized pre-transplant in decision-making regarding transplant eligibility.

### Immunotherapy and systemic targeted treatments for advanced-stage HCC

**Figure 4:**
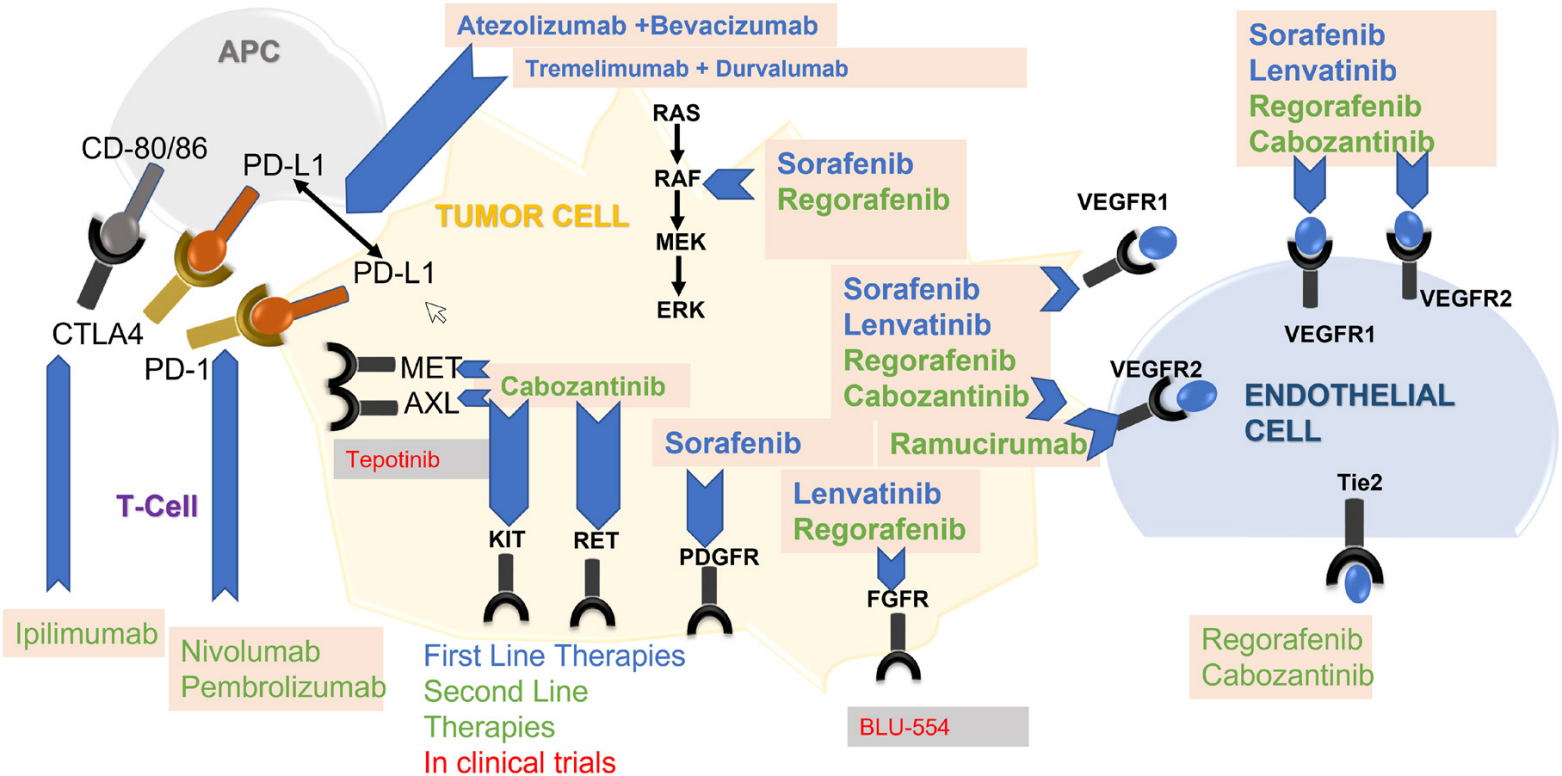
Molecular pathways and targeted therapeutics in hepatocellular carcinoma. Abbreviations: APC: antigen presenting cell; AXL is a cell surface receptor tyrosine kinase, part of the TAM family of kinases; CD: cluster differentiation; CTLA4: cytotoxic T-lymphocyte–assoc+iated antigen 4; ERK: extracellular signal-regulated kinase; FGFR: Fibroblast growth factor receptors; KIT: tyrosine-protein kinase; MEK: mitogen-activated protein kinase; MET: Mesenchymal Epithelial Transition; PDGFR: platelet-derived growth factor receptor; PD-L1: Programmed cell death ligand 1; RAF: rapidly accelerated fibrosarcoma; Tie2 is a receptor tyrosine kinase; VEGF: Vascular endothelial growth factor.

The immune component of the HCC microenvironment has been of great interest with the recent approval of immunotherapies, such as immune checkpoint inhibitors, for advanced stage HCC (BCLC Stage C) (Figure 4). However, only a subset of HCCs have high levels of immune cell infiltration, and the response rate of HCCs to immunotherapies is only around 10–35% [72, 73]. Thus, a key to the safe and effective use of such treatments will be correctly identifying patients most likely to exhibit a positive response.

Sorafenib is a multi-targeted tyrosine kinase inhibitor (TKI) that was the first approved target therapy and the standard of care for advanced HCC (Figure 4). Sorafenib exerts antiproliferative (*RAF1*, *MEK, ERK*), anti-angiogenic (*VEGFR2* and *PDGFR*), and pro-apoptotic effects [74]. The SHARP trial was a landmark study that led to its approval and demonstrated a modest survival benefit of sorafenib versus placebo (median OS 10.7 vs. 7.9 months, *P* < 0.001) [75]. Sorafenib is indicated as a first-line treatment option for patients with well-preserved liver function (Child-Pugh A) and advanced tumors (BCLC C) or tumors at an intermediate stage (BCLC B) that failed LDT.

The approval of Sorafenib in 2007 was followed by many largely unsuccessful late-stage trials assessing novel targeted therapies. However, starting in 2017, six additional systemic agents were approved for advanced-stage HCC, including for first-line therapy (lenvantinib) and second-line therapy (cabozantinib, regorafenib, and ramucirumab if AFP >400 ng/mL), as well as two immune checkpoint inhibitors (ICI) for second-line therapy (nivolumab and pembrolizumab) in the accelerated approval setting [76, 77]. However, this was followed by two recent phase 3 trials investigating the two approved anti-programmed death-1 (anti-PD-1) monoclonal antibody immunotherapies, nivolumab (1st line indication), and pembrolizumab (2nd line indication), that failed to reach their primary endpoints despite promising phase 2 results [78, 79].

In 2020, a breakthrough occurred in the landmark IMbrave150 study of atezolizumab (anti-PD-L1) + bevacizumab (anti-VEGF) versus sorafenib in advanced HCC [80]. This study demonstrated improved OS at 12 months of 67.2% vs. 54.6% with atezolizumab/bevacizumab and better progression-free survival (PFS) of 6.8 months vs. 4.3 months. This study required assessment and treatment of esophageal or gastric varices before enrollment due to the bevacizumab component. This study only included patients with preserved liver function with Child-Pugh A cirrhosis. A network meta-analysis of targeted immunotherapies also found superior OS with the combination of atezolizumab + bevacizumab used in the first-line setting compared to sorafenib, lenvatinib, and nivolumab [81]. The atezolizumab + bevacizumab combination is the first-line standard of care in patients with unresectable or metastatic HCC. Recently presented phase 3 data (HIMALAYA trial) with the novel immunotherapeutic combination of tremelimumab plus durvalumab demonstrated better OS and PFS compared to sorafenib [82]. The FDA approved this combination on Oct 21, 2022, making it an attractive first-line option in advanced HCC.

Current Treatment Landscape: The choice of therapy is dictated by hepatic synthetic function, portal hypertension, performance status and tumor burden. ASCO guidelines recommend atezolizumab-bevacizumab or TKIs (sorafenib/lenvatinib) as first line therapy. The choice of second-line therapy is dependent on initial treatment. Those treated with durvalumab-tremelimumab or atezolizumab-bevacizumab may be offered TKIs such as sorafenib, lenvatinib, regorafenib or cabozantinib. If the patient progresses despite sorafenib or lenvatinib as first line therapy and has contraindications to immune checkpoint inhibitors, regorafenib or cabozantinib could be an option, with ramucirumab utilized in those with AFP level more than 400 ng/mL. For those patients who progress on TKIs, dual immunotherapy (nivolumab-iplimumab ) is preferred as it has been shown to produce higher ORR. If unable to tolerate this combination, pembrolizumab monotherapy may be utilized as an alternative [83].

Despite these advances, there remain many other potential targets for HCC therapy. For example, the aberrant activity of the TGF-β pathway is involved in nearly 40% of HCCs, and deficiency of this pathway is observed in the cancer stem cell signature of HCC. Thus, this pathway represents a potential drug target. The JAK/STAT pathway is a signaling pathway that is aberrantly activated in some HCCs and represents a potential intervention target [84, 85]. Tumor sensitivity may differ between patients, so we can tailor radiation dose by biomarkers in the future (e.g., ADC, diffusion coefficient). The Cancer Genome Atlas (TCGA) genomics could provide vital clues in identifying specific groups that can be targeted with modalities such as radiation. For instance, TCGA analyses of 9,125 cancers, including HCCs, revealed that inactivation of the TGF-β pathway correlates with aberrant DNA crosslinking repair members and sirtuins and an increase in ‘Stemness’ in potentially 40% of HCCs. These HCCs may be more responsive to radiation therapy.

### Precision oncology in HCC

While personalized medicine has become a practical reality in other cancers, HCC has been difficult to target with specific treatments based on molecular profiling. One of the main reasons for targeted therapies’ failure is tumor heterogeneity (inter and intra-subject). A challenge to developing therapies is that somatic mutations occur in genes whose products are not easily or safely druggable, such as mutated forms of *TERT, TP53, CTNNB1*, and *MYC*. New strategies to target driver genes and pathways, such as micro-RNA-based therapeutics, are in development for other cancers and may apply to HCC in the future.

Neoantigens that arise from mutations in HCC tumors have emerged as important targets for future combinatorial immunotherapy [86–89]. However, most tumor mutations are not shared across individuals. Because of HLA diversity (i.e., the small proportion of shared neoantigens are recognized only by T cells of specific subsets of patients), immunogenic tumor neoantigens generally must be selected based on each patient’s tumor [90, 91]. Vaccines must also be customized. Nevertheless, several clinical trials conducted with patients with solid tumors and melanoma have demonstrated that personal neoantigen-directed vaccines are feasible, safe, and immunogenic [92–94]. Phase II trials are currently in progress for HCC harnessing this technology [87, 95].

Similar clinical trials are in progress using oncolytic viruses in combination with chemotherapy and immunotherapy [96, 97]. Recombinant oncolytic viruses harness the disparity of genetic mutations and molecular activity between liver cancer cells and normal cells, displaying a reasonable specificity for targeting HCCs. Oncolytic vaccinia virus such as JX-594 lacks the TK gene and expresses granulocyte-macrophage colony-stimulating factor (GM-CSF), specifically targets liver cancer cells with high cellular TK activity and activated EGFR signaling, which facilitates its replication [98].

### Novel targets for molecular therapies

In this section, we will briefly highlight promising, well-tolerated active compounds which target driver genes and pathways in HCC and are currently being studied for clinical application (Table 3).

**Table 3:** Molecular pathways and therapeutic targets in HCC

Targets	Therapeutic Agents	Phase
VEGFR, Ras/Raf/MEK/ERK, PDGFR, c-KIT, RET	Sorafenib	Approved
VEGFR	Ramucirumab	Approved
VEGFR, PDGFR, FGFR, RET, SCFR	Lenvatinib	Approved
VEGFR, PDGFR, BRAFFGFR, KIT, RET	Regorafenib	Approved
EGFR/ErbB1/Her1	Erlotinib	3
PI3K/Akt/mTOR	Everolimus	3
PI3K/Akt/mTOR	Sirolimus	3
c-MET	Cabozantinib	Approved
TGF-β1 Receptor Type I	Galunisertib	2
Fibroblast Growth Factor 4 (FGFR4) inhibitor	BLU-554 (fisogatinib)	1
Tyrosine kinase receptor c-MET (MET)	Tepotinib	2

### MET inhibitors

The tyrosine kinase receptor c-MET (MET) through its ligand, hepatocyte growth factor (HGF) leads to HCC progression by promoting cellular proliferation and invasion, as well as by mediating tyrosine kinase resistance. The clinical utility of older MET inhibitors had been limited by their toxicity. However, newer and more selective oral compounds have demonstrated promising results with good tolerability. Recent phase 1b/2 trials of tepotinib (FDA-approved for non- small cell lung cancer) in treatment naïve Asian patients (https://clinicaltrials.gov/ NCT01988493) [99] and in sorafenib resistant non-Asian cohorts with HCC (https://clinicaltrials.gov/: NCT02115373) [100] showed that 63.3% of patients were progression-free at 12 weeks. However, a low overall response rate (ORR of 8%) and disease control rate (DCR of 27%) suggest that selective MET inhibition is effective in only a minority of even those patients selected on the basis of MET positivity on immunochemistry [100].

### Fibroblast growth factor (FGF) receptor inhibitors

The FGF family and its receptors (FGFR) are involved in multiple carcinogenic pathways. Aberrant FGF/FGFR signaling has been noted to enhance HCC cell invasion by suppressing E-cadherin expression and promoting the expression of epithelial-to-mesenchymal transition-related genes. BLU-554 (also known as fisogatinib) is the most advanced of the clinically translatable FGFR4 inhibitors and is still being investigated in a phase I study for the treatment of advanced HCC (https://clinicaltrials.gov/ Identifier: NCT02508467). Another compound being studied in a phase 1 study for advanced HCC is H3B-6527 (https://clinicaltrials.gov/ Identifier: NCT02834780). FGF401 (also known as roblitinib) is a reversible covalent inhibitor, displaying more than a 100-fold selectivity for FGFR4 compared to FGFR1-3. FGF401 exhibits potent antitumor activity in HCC with aberrant FGF19 overexpression. Novartis pharmaceuticals sponsored a clinical trial of FGF401 from 2014 to 2020. This study aimed to test the maximum tolerated dose and/or recommended phase II dose and efficacy of FGF401 as a monotherapy or in combination with PDR001 in HCC patients positive for FGFR4 and KLB (https://clinicaltrials.gov/ Identifier: NCT02325739). This study has been completed but conclusive data have not been published, and the phase II part of the FGF401 + PDR001 combination was halted due to commercial reasons.

Galunisertib (LY2157299 Monohydrate), a TGF-β receptor 1 inhibitor which had shown promise in sorafenib resistant HCC, has also been withdrawn from clinical use. From these therapeutic trials, we may conclude that molecular therapy for HCC has potential, but is fraught with challenges.

### Disparities in HCC incidence and mortality are prevalent

Significant disparities in HCC incidence and mortality due to geographic location, sex, race, ethnicity, sociocultural contexts, and clinical factors exist. Globally, liver cancer incidence and mortality rates are double to triple in men compared to women [1]. High-risk areas for HCC include China, the Republic of Korea, and sub-Saharan Africa [1]. Within the U.S., surveillance data indicate widespread racial and ethnicity-based liver and intrahepatic bile duct cancer disparities [101]. Liver cancer incidence and mortality are highest in the American Indian or Alaska Native (AI/AN) population, with an almost two-fold higher rate than Whites in the most recent data surveys (2014–2019) [102]. Similarly, liver cancer incidence and mortality are also very high for the Hispanic and Asian/Pacific Islanders [102]. Additionally, US-born Hispanic individuals have been reported to have higher HCC incidence compared to foreign-born individuals [103]. These differences can partially be explained by the geographical variation in the prevalence of the known HCC risk factors. Countries with high HBV or HCV prevalence also had high HCC incidence [104]. The high prevalence of HCC in Asian individuals in the U.S. can also be explained by a high number of foreign-born individuals from countries with high HBV prevalence [105, 106]. In the US, there is evidence of a higher rate of metabolic disorders related to HCC etiology in Hispanics and higher rates of chronic infection with HBV or HCV in Hispanic and African American men [107, 108].

Variability in clinical factors, including access to and quality of healthcare, also account for disparities in HCC incidence and mortality. Hispanic and Black HCC patients are less likely to be diagnosed early or undergo curative treatment than white patients [109]. Black or Hispanics with early-stage HCC living in counties with poor social determinants of health (SDOH) are less likely to receive surgical intervention than in other counties [110]. A similar dichotomy exists between rural and suburban dwellers, with rural liver cancer patients less likely to undergo treatment resulting in higher rural mortality due to liver cancer compared to patients in more urban and peri-urban areas [111].

While differential incidence and mortality of HCC in different populations are likely caused by differential exposure to infectious agents, the prevalence of metabolic syndrome and chronic liver disease, and disparities in health care quality, the role of genetic and other molecular mechanisms cannot be ruled out. For example, the mutational frequency of *TP53* in HCC in the TCGA cohort is significantly different between different racial and ethnic groups (Black or African Americans, 70.6%, Asians, 36.5%, and Caucasians, 22.8%) [112], suggesting differential tumor drivers according to the population of origin. Further studies examining the genetic and biological basis of race and ethnicity-based HCC disparities are needed to determine tumor biological factors that account for the unexplained causes of HCC disparities at the molecular level, such that interventional strategies can be designed appropriately. Awareness of these disparities and their causes should influence HCC patient management by focusing on interventions that can contribute to addressing inequities in cancer prevention, screening, and treatment in HCC.

## FUTURE DIRECTIONS

While surgical therapies can be curative, only a small proportion of patients with HCC will qualify. Despite early detection and chemoprevention efforts, HCC incidence continues to arise, frequently in more advanced stages. The prognosis of intermediate to advanced HCC remains poor, and there is much room for improvement. Research through histological classification, whole genome characterization, and the development of multiple viable animal models has led to substantial progress on the pathogenesis of HCC over the last decade. This has culminated in the approval of multiple new treatments for HCC. However, there are several significant challenges to future progress in this complex malignancy. The limited tissue specimens for measuring biomarkers discovered through publicly-available databases for HCC remains a major issue. The absence of clinically validated commercially available biomarkers with high sensitivity and specificity is an important reason why HCC diagnosis and treatments have lagged decades behind other solid cancers. There remains an urgent need for integrating biomarkers found in HCC into clinical practice. In addition, diversifying the study and clinical trial cohorts for adequate representation of racial and ethnic groups disproportionately affected by HCC is critical to ensure that biomarkers developed and personalized medicine approaches will be pertinent in these populations. Successfully harnessing integrated approaches that combine bioinformatics analysis with *in vivo* animal models and *in vitro* biological and biochemical methods is the key to effectively developing future cures for this lethal cancer.

## Abbreviations

HCC: hepatocellular carcinoma
IACS: interaction analysis
GEM: genetic and epigenetic mechanisms
US: ultrasound
CT: computed tomography
MRI: magnetic resonance imaging
HCV: hepatitis C virus
HBV: hepatitis B virus
ETOH: ethanol alcohol
